# Inoculation with arbuscular mycorrhizal fungi can increase the concentration of per and poly-fluoroalkyl substances in cereal crops

**DOI:** 10.1007/s00572-026-01276-1

**Published:** 2026-06-03

**Authors:** Stephanie J. Watts-Williams, Sara Thomas, Alison R. Gill, Thi Diem Nguyen, Shervin Kabiri

**Affiliations:** 1 Centre for Crop Systems Analysis, Wageningen University & Research, Wageningen, 6708 PE The Netherlands; 2 The Waite Research Institute, The School of Agriculture, Food and Wine, Adelaide University, Waite Campus, Urrbrae, South Australia Australia; 3 Australian Research Council Centre of Excellence in Plants for Space, Adelaide, South Australia Australia; 4 Institute of Biotechnology, Hue University, Phu Thuong Ward, Thuan Hoa District, Hue City, Vietnam

**Keywords:** Durum wheat, Barley, Bread wheat, PFAS, Soil contamination

## Abstract

Contamination of agricultural soils with per- and poly-fluoroalkyl substances (PFAS) is now widespread and common throughout the world due to management practices such as biosolid application. Crop and pasture plants can readily take up and accumulate PFAS in their tissues, which eventually end up in food products or livestock feed and lead to health issues. This study investigated how arbuscular mycorrhizal (AM) fungi affect plant PFAS uptake in three important cereal crop species (barley, bread wheat, durum wheat), using a combination of soils that were artificially spiked or naturally contaminated with PFAS. Spiking soil with PFAS did not interfere with AM colonisation of roots, nor did it detrimentally affect plant biomass. However, the shoots of all three plant species accumulated high concentrations of multiple short- and long-chain PFAS compounds. Inoculation with AM fungi led to increased concentration of all measured PFAS compounds in durum wheat shoots, the highest being PFBS at 5.2 ng g^− 1^. Several compounds were also significantly greater with AM fungal inoculation in barley shoots. The uptake of PFAS in cereal plants is highly dependent on the nature of the soil PFAS contamination (i.e., the concentration and speciation), and the plant species in association with AM fungi. It remains to be investigated whether AM fungi can directly take up PFAS from the soil.

**Supplementary Information:**

The online version contains supplementary material available at 10.1007/s00572-026-01276-1.

## Introduction

Per- and poly-fluoroalkyl substances (PFAS) are a group of highly stable chemicals that are common components of household and industrial products including adhesives, coatings, packaging, and pesticides since the 1940s (Wang et al. 2020). PFAS do not easily degrade in the environment and persist in terrestrial and aquatic systems alike. This allows PFAS to easily enter the food chain at several different points in food production systems, bioaccumulating in plants, livestock, and eventually humans. The accumulation of PFAS in human bodies can lead to serious health issues, including immune and endocrine disorders, cardiovascular disease, and reproductive and developmental disruptions (Jha et al. 2021). The cost of PFAS contamination in the food chain to the health system is estimated at €52–84 billion per annum in Europe alone (Cordner et al. 2021), so the mechanisms for PFAS uptake from soil and water into food crops must be uncovered to devise ways to reduce or block bioaccumulation at this step in the food chain.

Agricultural soils become contaminated with PFAS through a number of pathways including contaminated irrigation water, application of organic matter to the soil as an alternative to conventional fertilisers (e.g., sewage sludge, biosolids), leaching from landfill, and pesticide use (Wang et al. 2020). Plants, including many popular food crops, readily take up the small and water-soluble PFAS via their roots, and accumulate them in shoots and grains (Adu et al. 2023). A study by Ofoegbu et al. (2022) in wheat plants grown in soil spiked with perfluorooctane sulfonic acid (PFOS) to different concentrations found that, although PFOS contamination did not affect the yield of the plants, PFOS accumulated substantially in the wheat grains. Meanwhile, Wen et al. (2014) conducted a field study that evaluated increasing applications of biosolids on the uptake and translocation of PFAS in wheat plants, and reported that the transfer factors of PFAS compounds from the straw to the grain was higher for perfluorosulfonic (PFSA) than perfluorocarboxylic (PFCA) acids.

A relatively unexplored avenue for the uptake of PFAS by plants is that of arbuscular mycorrhizal (AM) fungi. AM fungi are ubiquitous in agricultural soils (Frew et al. 2025), and benefit plant growth by acquiring inorganic nutrients and other solutes from the soil and transporting them into the plants via structures in the root cortical cells called arbuscules (Smith and Read 2008). Several studies showed that AM fungi can reduce the uptake of soil contaminants such as heavy metals into the host plant, conferring a positive effect on plant biomass compared to a non-colonised control plants (Chen et al. 2003; Watts-Williams et al. 2013). This is known as a ‘protective’ function of AM fungi that contrasts with the function of soil nutrient uptake that the association is typically reported in the literature.

The literature on AM fungal interactions with PFAS are limited, with some reporting positive and some reporting negative consequences for the host plant. One study found that soil perfluorooctanoic acid (PFOA) contamination led to reduced AM colonisation in spring onion plants, and negatively affected plant performance (as biomass and root morphology) (Yan et al. 2025). The authors concluded that inoculation of the plants with AM fungi may have reduced the negative effect of PFOA contamination on the host plant, through alterations to resource allocations strategy that favours shoot growth. A very recent study found that AM fungi play multiple roles in the transformation of PFAS in soil and the removal of PFAS from the soil (Wang et al. 2025); this included the promotion of plant (wetland species *Iris pseudacorus*) uptake of PFAS and increased concentration of PFAS in plant tissues. Another study from the same research group found that AM colonisation increased the tolerance of their host plant to oxidative stress from PFAS exposure, including promotion of plant growth and photosynthetic efficiency (Wang et al. 2024).

The effect of AM fungi on PFAS concentrations in crop plants has not yet been explored. Given there is limited existing data, there are three possible scenarios regarding how AM fungi affect plant PFAS concentrations. There may be no effect (neutral), or the AM fungi may ‘protect’ the host plant and reduce the concentration of PFAS in shoots, or conversely, they may promote host plant uptake of PFAS from the soil. Once we have an understanding of how AM fungi affect plant PFAS uptake, we can target future research to best manage AM fungi and soil biology more broadly, while minimising PFAS uptake into crops. This may be done through introducing new species of AM fungi into fields that best immobilise PFAS to prevent plant uptake, by selecting crop varieties that do not associate with AM fungi to suppress PFAS uptake, or by using AM fungi to facilitate plant-based strategies for soil remediation (phytoextraction) (Kavusi et al. 2023).

The following experiments had the overarching aim to determine the effect of soil PFAS contamination and AM fungi on three important cereal crops (barley, bread wheat, and durum wheat), with the specific aims to determine:

i. Whether there is interspecific variation in plant response to PFAS contamination;
ii. Whether soil PFAS contamination affects AM fungal colonisation of roots; and
iii. Whether inoculation with AM fungi increases resilience of host plants to PFAS contamination in the soil.

## Methods

### Soil preparation and AM fungal inoculation

The soils used in Expt. 1 were a sandy clay loam soil collected from a non-contaminated area in South Australia (Soil 1), and a sandy soil from an aqueous film-forming foam (AFFF)-contaminated area (Soil 2) due to the repetitive application of the AFFF over time. Soil 1 had no PFAS contamination; half of Soil 1 was spiked with AFFF to a ∑PFAS of 6.9 mg kg^− 1^ and the other half was not spiked (control). Soil 2 had an existing ΣPFAS of 1.9 mg kg^− 1^ soil, which comprised different types of PFAS (Table S1. Supporting Information) while PFOS was the dominant compound (1.76 mg kg^− 1^).

Soil 1 had a pH (in water) value of 8.3 and Soil 2 had a pH of 7.2. Plant-available (Colwell 1965) phosphorus (P) was < 5 mg P kg^− 1^ for both soils, nitrate was 6.7 and 1.0 mg N kg^− 1^, and ammonium < 1 and 2.1 mg N kg^− 1^.The effective cation exchange capacity of Soil 1 was 19.3, and Soil 2 was 1.28. Soils were sieved to < 2 mm and then Soil 2 was mixed with a fine beach sand to dilute the existing PFAS concentration, while Soil 1 was mixed approximately 50:50 *w/w* with the fine beach sand to match the texture of the Soil 2. The final concentration of PFAS in the spiked soil (Soil 1) was greater than the AFFF-contaminated soil (Soil 2), while the unspiked soil (Soil 1) had no PFAS. Each 100 mm pot held 500 g of soil/sand mix.

### Experiment 1 – supplementation of the AM fungal community with a commercial inoculant

To test the effect of introduced AM fungi to field soils containing the native AM fungal community, both Soil 1 and Soil 2 were inoculated with a commercial product from MicrobeSmart (StartUp Ultra, Adelaide, Australia) that contained four isolates of the AM fungus *Rhizophagus irregularis*. Average AM fungal spore number was estimated by sieving and microscopy, and the product was added at a rate of ~ 400 spores plant^− 1^ to the + AMF treatment soil. No spores were added to the non-inoculated (control) soil.

### Experiment 2 – inoculation of sterilised soil with AM fungi

For this experiment, Soil 1 was autoclaved two times prior to inoculation with AM fungi (conducted as for Expt. 1). This allowed us to test the effect of AM fungal inoculation on plant PFAS accumulation against a truly non-mycorrhizal control (i.e., no native AM fungi present). While autoclaving may alter soil physicochemical properties, in this study, the soil that was sterilised and then spiked was a sandy soil with low organic carbon content. We expect that the effect of the autoclave would be negligible on changes in the soil properties. Our previous work on 12 different soils (Kabiri et al. 2022) demonstrated that PFAS readily leach from sandy soils with low/high organic carbon content, even at relatively high PFAS concentrations. This suggests that PFAS retention in such soils is minimal, and therefore the effects of autoclaving on sorption/desorption behaviour are expected to be negligible.

### Plant preparation and growth conditions

In both experiments, three species of cereal crop were included: *Hordeum vulgare* (barley) var. Compass, *Triticum aestivum* (bread wheat) var. Scepter, and *Triticum durum* (durum wheat) var. Westcourt. The species and varieties were selected after consultation with the relevant industry, based on their popularity in Australian agriculture. For both experiments, all seeds were surface sterilised in 10% bleach solution for 10 min, rinsed well with RO water, then moved onto moist filter paper and sealed in petri dishes, before being left at room temperature under laboratory lighting to germinate, with occasional watering when the filter paper was almost dry. Three days later, the germinated seedlings were transplanted to the prepared soils. There were five biological replicates of each treatment.

All plants were grown in a controlled environment chamber where the day and night temperatures were 24 °C and 18 °C, respectively, and daylength was set at 16 h. Pots were arranged in a randomised complete block design with five biological replicates per treatment. Plants were watered 3–4 times per week to 90% of field capacity, with no water (and thus PFAS) being allowed to leach from the soil. Plants were given 10 mL of full-strength Long-Ashton nutrient solution on three occasions during the growing period at 19, 33 and 47 days after planting.

### Harvest and sample analysis

Plants were destructively harvested 73 days after transplantation to the soils. Roots were washed free of soil using RO water, then shoots and roots were separated and weighed. A subsample of fresh roots of approximately 200 mg was taken from each plant and stored in 50% ethanol solution for later root staining and AM fungal colonisation quantification. After sub-sampling, the roots were weighed again and then the root and shoot samples were placed in 25 mL plastic sample jars and covered with a Kimwipe secured by rubber band, in preparation for freeze-drying. All fresh plant samples were then freeze dried for 72 h at -20 °C. Following drying, the plant samples were weighed again to obtain shoot and root dry weight data.

### Plant PFAS analysis

PFAS was extracted from the plant tissues following modified methods by Nassazzi et al. (2022). The plant samples were freeze dried and then 0.1 g of the dried samples homogenised in ball mill (Retsch) using a zirconium jar and balls using three balls in 100 mL jar. Each milling was conducted for 30 s at 500 rpm and after milling the jar and balls were washed with water first followed by LC-MS grade methanol to avoid any PFAS contamination to next samples. All samples were spiked with PFAS stable isotope solution at 5 ng mL^− 1^ in the final extract. The sample was vortexed and equilibrated for 30 min prior to extraction, then extracted three times with 4 mL of methanol containing 0.1 M of ammonium hydroxide (NH_4_OH). Each extraction consisted of 5 min of vortex followed by centrifugation for 10 min at 2500 g. Supernatant from the three extractions were combined and evaporated under N_2_ at 40 °C using a Dry Block Multivap Evaporator to 1 mL. The extracts were then passed through an solid phase extraction (SPE) cartridge (Agilent, Bond Elut Carbon) which was pre-conditioned with methanol. The final eluent was collected into a polypropylene autosampler vial, blown down under N_2_ and made to a final volume of 1 mL. A spiked control sample and a method blank were extracted alongside each batch of samples.

Chromatography was performed on an Agilent 6495 liquid chromatography/triple quadrupole mass spectrometer (LC/TQ) system. The separation of PFAS compounds was done on an Agilent ZORBAX RRHD Eclipse Plus C18 (2.1 × 100 mm, 1.8 μm), with an Agilent guard column ZORBAX RRHD Eclipse C18 (2.1 mm, 1.8 μm), equipped with an InfinityLab PFAS delay column, 4.6 × 30 mm. Mobile phases were 2 mM of ammonium acetate in 95% water, 5% acetonitrile (A) and 100% acetonitrile (B). Injection volume was 5 µL (Agilent 1290 Infinity II) and the flow rate was 0.4 mLmin^− 1^. The column oven was kept at 40 °C and the autosampler at 10 °C. The solvent gradient and LC/TQ instrument parameters are provided in Table S2and S3 of Supporting Information. Negative electrospray ionization was used with calibration range of 0.01 to 100 ng mL^− 1^. All standards contained the same 13 C PFAS concentration as the samples for each run. Every 10 to 15 samples, a solvent blank and a standard solution were analysed to track instrument performance. Calibration curves weighed 1/x, automated peak integration was used however, integrations were manually curated to ensure accuracy.

Agilent 6495 LC/TQ with Agilent Jet Stream (AJS) electrospray ion source was operated in dynamic multiple reaction monitoring (dMRM) mode. This allows the addition of more MRM transitions for additional compounds if needed. The LC/TQ autotune was performed and all data acquisition and processing were performed using the Agilent MassHunter software version 10.

### AM colonisation analysis

Root samples preserved in ethanol were rinsed thoroughly with RO water before being placed in a 10% KOH solution for seven days at room temperature. Cleared roots were rinsed well with RO water before being submerged in a 5% ink in vinegar solution at 60 °C for 15 min (Vierheilig et al. 1998). The stained roots were subsequently rinsed thoroughly and allowed to de-stain in a 5% vinegar solution for one day, before being moved to a 50% glycerol solution for storage and microscope analysis. Root length colonised by arbuscular mycorrhizal fungi was quantified by the gridline intersect method (Giovannetti and Mosse 1980). The stained root subsample was spread over a 100 mm Petri dish lined with transparent 10 mm gridline paper, and at each intersection of a root and line, it was determined whether the root was colonised by AM fungi or not. Once 150 intersections had been recorded, a percentage of root length colonised was calculated for each sample.

### Statistical analysis

All data were checked for normality using the Shapiro-Wilk test. Any variables that did not conform to the assumption of normality (*P* > 0.05) were analysed using the Kruskal-Wallis test instead of Student’s t-test.

Expt. 1 plant data included AM colonisation, biomass and shoot PFAS concentrations (sulfonic acids), which were analysed by two-way ANOVA where the factors were *Mycorrhiza* and *Soil*, for each plant species, respectively. Significant main effect or interaction terms were further interrogated using Tukey’s post hoc test. Only plants from the PFAS-contaminated soils (Soil 1 spiked and Soil 2) were analysed for shoot PFAS concentrations (sulfonic acids), and a Student’s t-test was employed for each compound to compare concentrations in the AM-inoculated vs. non-inoculated plants of each plant species, respectively.

Expt. 2 plant biomass data were analysed by two-way ANOVA with the factors *Mycorrhiza* and *Soil*; only the spiked soil plants were analysed for tissue PFAS concentrations (sulfonic and carboxylic acids), and Student’s t-test was employed for analysis, with *Mycorrhiza* as the factor. All statistical analyses were conducted and graphs plotted using R Statistical Software.

## Results

### Experiment 1

#### AM colonisation

**Fig. 1:**
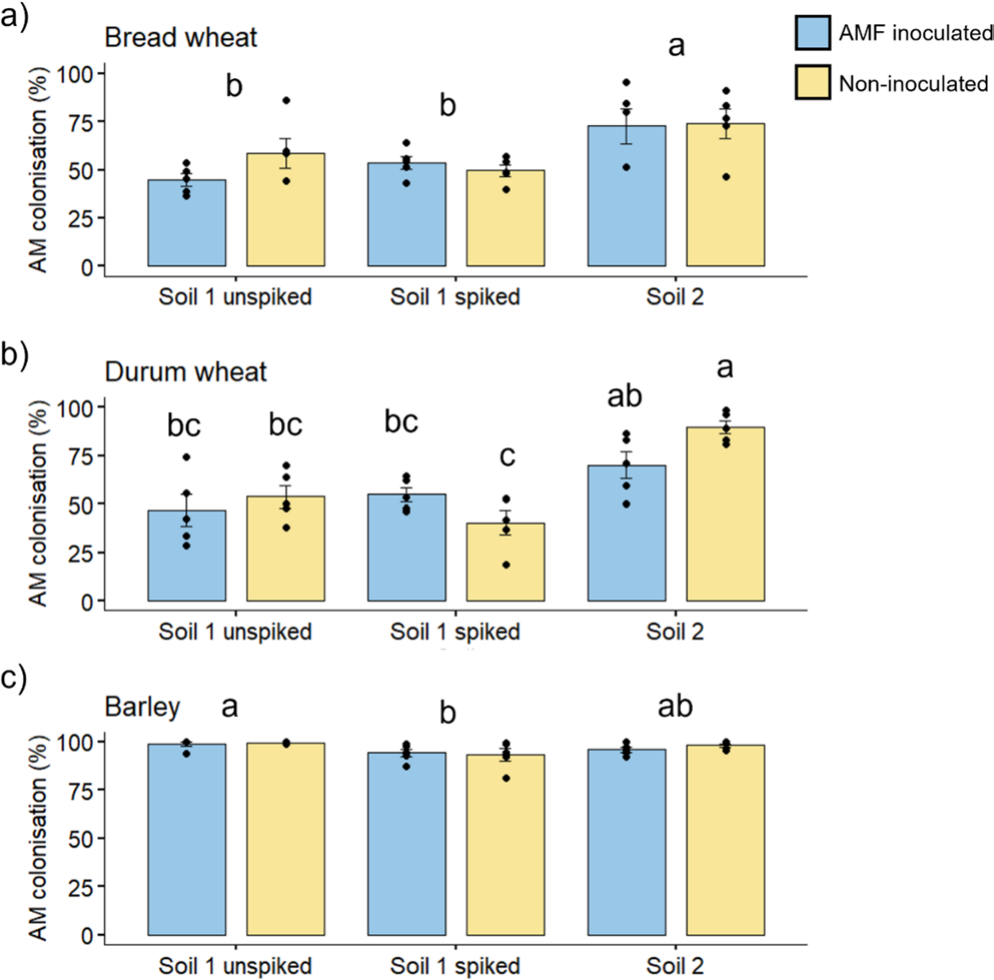
Percentage of root length colonised by arbuscular mycorrhizal (AM) fungi in bread wheat (**a**), durum wheat (**b**) and barley (**c**), either grown in Soil 1 unspiked, or spiked with PFAS, or Soil 2, naturally contaminated with PFAS, and inoculated (blue) or not (yellow) with a commercial source of the AM fungus *Rhizophagus irregularis* (Experiment 1). Bars are treatment mean ± standard error of the mean, *n* = 5. Letters indicate differences following Tukey’s HSD *post hoc* test, where means that share a letter are not significantly different. Figures with three sets of letters indicate a significant main effect of *Soil*, while figures with six sets of letters indicate a significant interaction between *Soil* and *Mycorrhiza* in the ANOVA (see Table S2 Supporting information for further detail of statistical outcomes)

All the plants were well-colonised by AM fungi, with root length colonised values ranging from 40.4% (durum wheat, Soil 1 spiked) to 99.3% (barley, Soil 1 unspiked) (Fig. 1). In this study, there was little evidence of a detrimental effect of soil PFAS on AM colonisation of roots – only in barley, with a small but significant reduction in colonisation percentage. For bread wheat, the plants grown in Soil 2 were more colonised by AM fungi than in Soil 1 (see ANOVA outcomes in Table S4 Supporting information). In durum wheat, a similar result was observed, where the plants grown in Soil 2 were generally more colonised by AM fungi than in either treatment with Soil 1. In barley, root AM colonisation was effectively saturated across all treatments (mean value for barley was 96.6% root length colonised), but AM colonisation was significantly higher in the unspiked Soil 1 plants compared to the spiked Soil 1 plants.

#### Plant biomass

**Fig. 2:**
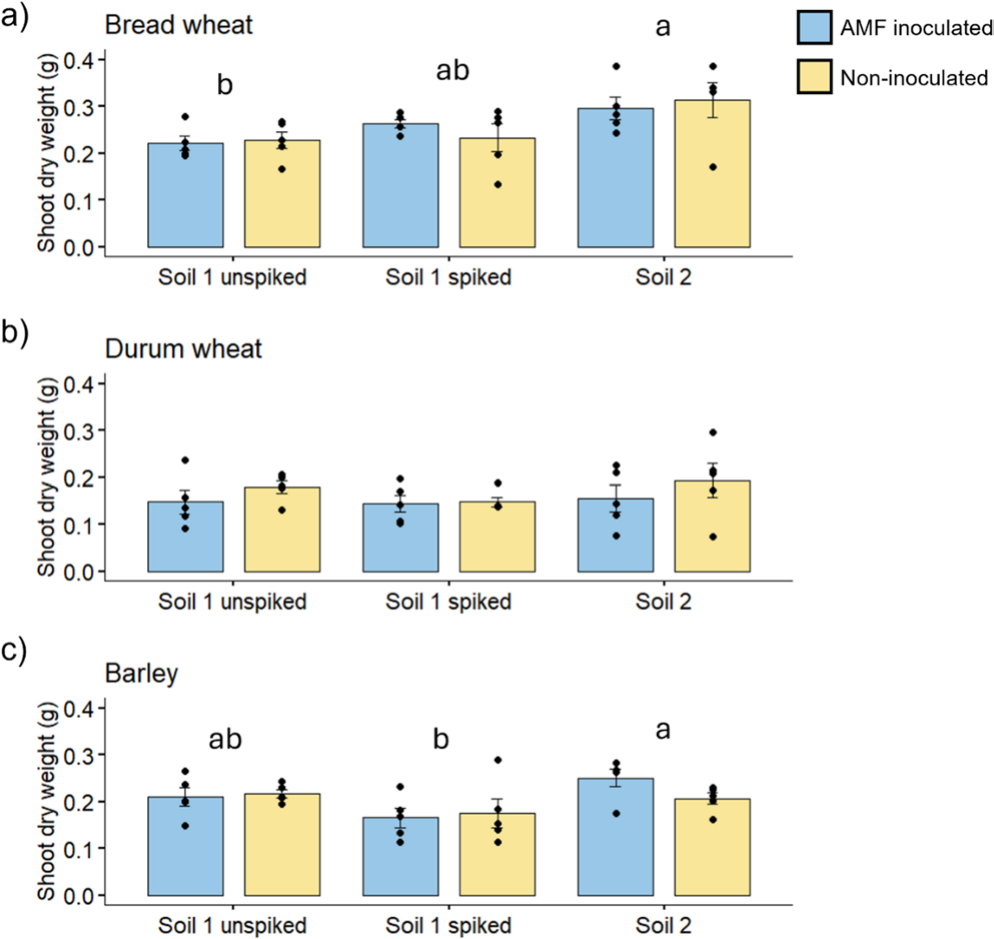
Shoot dry weights (g) of bread wheat (**a**), durum wheat (**b**) and barley (**c**), either grown in Soil 1 unspiked, or spiked with PFAS, or Soil 2, naturally contaminated with PFAS, and inoculated (blue) or not (yellow) with a commercial source of the AM fungus *Rhizophagus irregularis* (Experiment 1). Bars are treatment mean ± standard error of the mean, *n* = 5. Letters indicate differences following Tukey’s HSD *post hoc* test, where means that share a letter are not significantly different. Figures with three sets of letters indicate a significant main effect of *Soil* in the ANOVA (see Table S2 Supporting information for further detail of statistical outcomes)

In the bread wheat and barley plants, shoot biomass was affected by the Soil treatment only (Fig. 2), where the plants grown in Soil 2 produced more shoot biomass than the respective crop when grown in Soil 1. In bread wheat, the plants in Soil 2 experienced a 25.3% increase in biomass compared to Soil 1 (mean across spiked and unspiked treatments), and in barley, there was a 17.2% increase in biomass in Soil 2. The durum wheat plants also displayed a small increase in biomass in Soil 2, but this was not statistically significant. There were no significant differences in biomass between the unspiked and spiked Soil 1.

#### Shoot PFAS concentrations

**Fig. 3:**
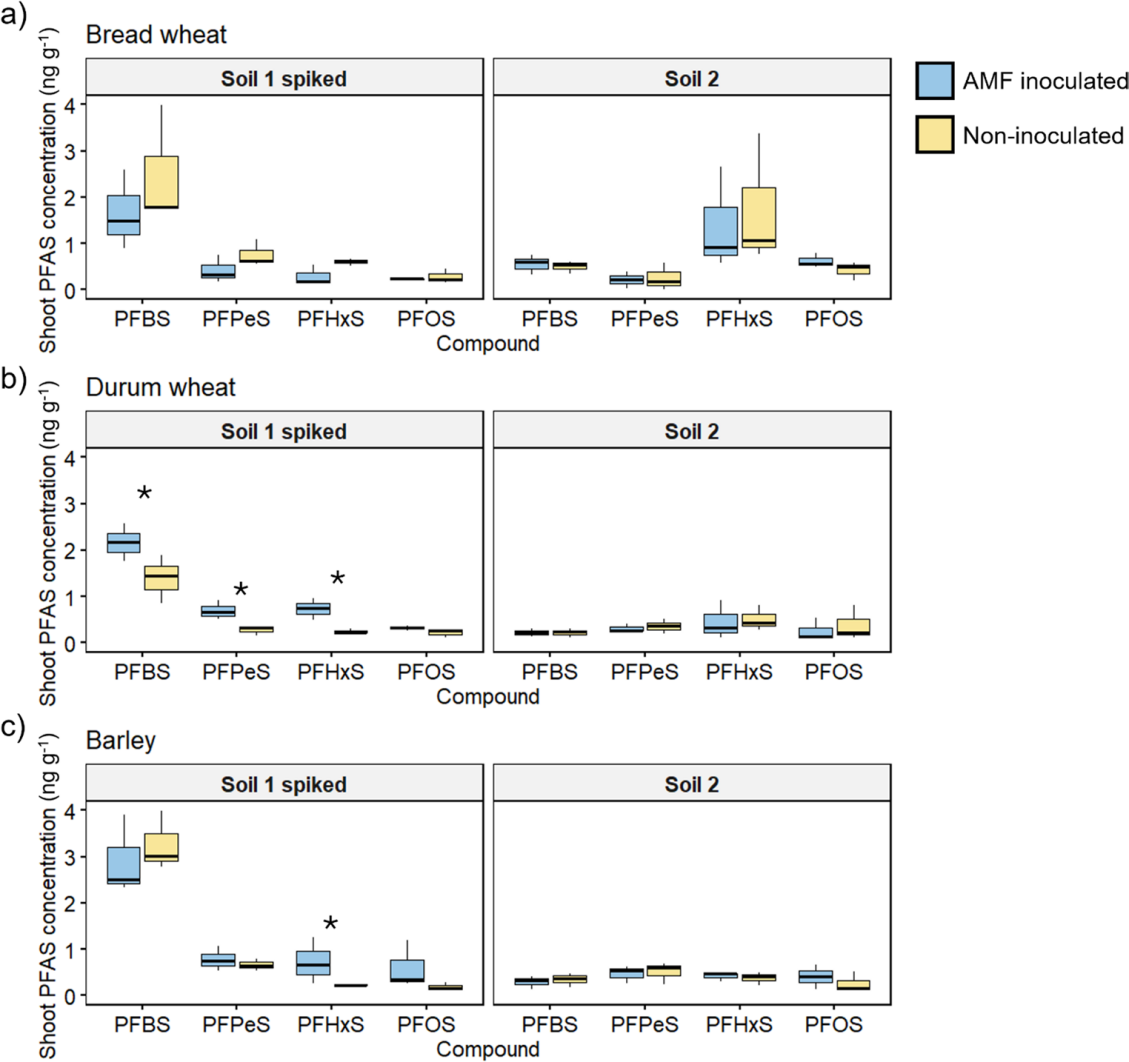
Concentration (ng g^− 1^) of four sulfonic PFAS in the shoots of bread wheat (**a**), durum wheat (**b**) and barley (**c**), either grown in Soil 1 spiked with PFAS, or Soil 2, naturally contaminated with PFAS, and inoculated (blue) or not (yellow) with a commercial source of the AM fungus *Rhizophagus irregularis* (Experiment 1). Boxplots indicate the minimum, interquartile ranges, median (thick line), and maximum value, *n* = 3. Asterisks indicate a significant difference in treatment mean between the AMF-inoculated and non-inoculated plants for an individual sulfonic acid, following Student’s *t*-test

The Experiment 1 plants were analysed for four perfluorosulfonic acid compounds: PFBS, PFPeS, PFHxS and PFOS. In this study, PFAS speciation refers to the identification and quantification of individual PFAS compounds with different chain lengths and functional groups (e.g., perfluoroalkyl carboxylic acids and sulfonic acids), which is essential for understanding their environmental behaviour, transport, and bioaccumulation. The concentration and the speciation profile of sulfonic acids in the plants was highly influenced by the soil they were grown in, either spiked with PFAS solution (Soil 1) or a PFAS contaminated soil (Soil 2) collected from the field (Fig. 3).

The durum wheat plants grown in the PFAS-spiked Soil 1 had greater shoot concentrations of PFBS, PFPeS and PFHxS when inoculated with the AM fungus *R. irregularis.* For example, shoot PFHxS concentration was mean 0.73 ng g^− 1^ in the inoculated durum wheat plants, compared to mean 0.25 ng g^− 1^ in the non-inoculated plants. In comparison, the durum wheat plants grown in Soil 1 that had not been spiked with PFAS had a mean PFHxS concentration of 0.01 ng g^− 1^ (Table S5 Supporting information). The barley plants had greater PFHxS concentration when inoculated with *R. irregularis*, but there were no significant effects of AM fungal inoculation on bread wheat.

In general, all three crop species accumulated higher concentrations of PFAS compounds in the spiked soil (Soil 1) than the naturally contaminated soil (Soil 2). In Soil 1, the PFAS compounds displayed the expected trend of higher concentrations of shorter chain compounds, but this was not seen in the plants grown in Soil 2, where PFBS concentration was low compared to the other measured compounds.

### Experiment 2

#### AM colonisation

**Fig. 4:**
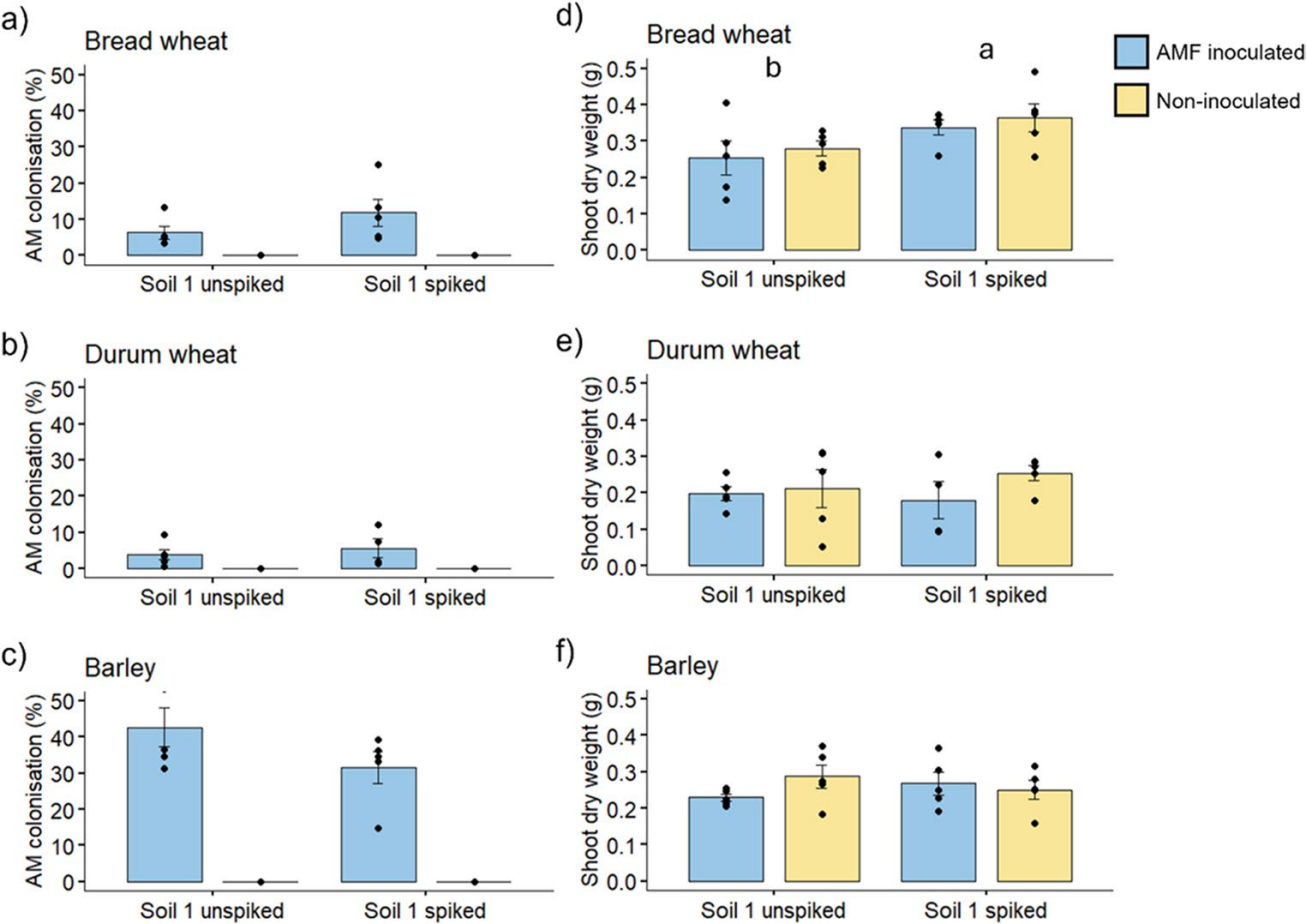
Percentage of root length colonised by arbuscular mycorrhizal (AM) fungi, and shoot dry weights of bread wheat (**a**, **d**), durum wheat (**b**, **e**) and barley (**c**, **f**), either grown in Soil 1 unspiked, or spiked with PFAS, and inoculated (blue) or not (yellow) with a commercial source of the AM fungus *Rhizophagus irregularis* (Experiment 2). Bars are treatment mean ± standard error of the mean, *n* = 5. Letters indicate differences following Tukey’s HSD *post hoc* test, where means that share a letter are not significantly different. Figures with two letters indicate a significant main effect of *Soil* in the ANOVA

In the second experiment, the plants were grown in Soil 1 that had been sterilised by autoclaving, and then was either re-inoculated with a commercial source of the AM fungus *R. irregularis*, or not inoculated. Thus, the extent of AM colonisation was greatly reduced in Experiment 2 compared to Experiment 1 which used Soil 1 that had not been sterilised. For example, in barley, the mean colonisation was 37.1% in Expt. 2, compared with 96.5% in the equivalent soil from Expt. 1 (Fig. 4a). The bread wheat and durum wheat plants experienced relatively low AM colonisation (mean 9.1% and 4.6% root length colonised, respectively) in the sterilised and re-inoculated Soil 1. In the sterilised, non-inoculated soil, none of the plants showed any evidence of AM colonisation under the microscope, confirming that the soil sterilisation process was effective.

#### Plant biomass

As with Expt. 1, the shoot biomass was not affected by AM colonisation in any of the three crop species (Fig. 4b). For bread wheat, the shoot biomass increased significantly (an increase of 27%) when grown in the PFAS-spiked soil compared to the unspiked control soil. A similar but non-significant trend was observed in the bread wheat grown in Expt. 1 (increase of 10% in the spiked Soil 1). This “fertilisation effect” of the PFAS spiking of Soil 1 was not observed in the durum wheat or the barley.

#### Shoot PFAS concentrations

**Fig. 5:**
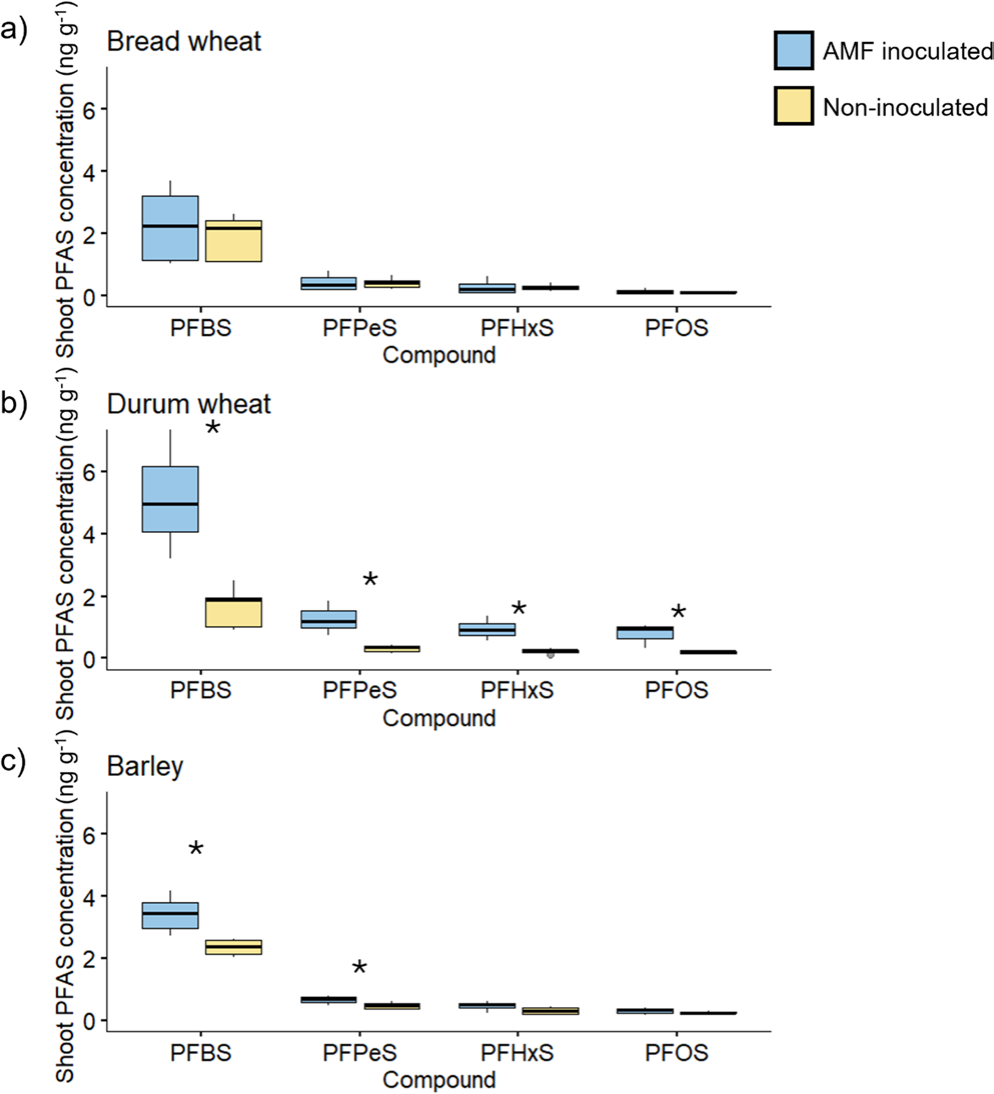
Concentration (ng g^− 1^) of four sulfonic PFAS in the shoots of bread wheat (**a**), durum wheat (**b**) and barley (**c**), grown in Soil 1 spiked with PFAS, and inoculated (blue) or not (yellow) with a commercial source of the AM fungus *Rhizophagus irregularis* (Experiment 2). Boxplots indicate the minimum, interquartile ranges, median (thick line), and maximum value, *n* = 5. Asterisks indicate a significant difference in treatment mean between the AMF-inoculated and non-inoculated plants for an individual sulfonic acid, following Student’s *t*-test

**Fig. 6:**
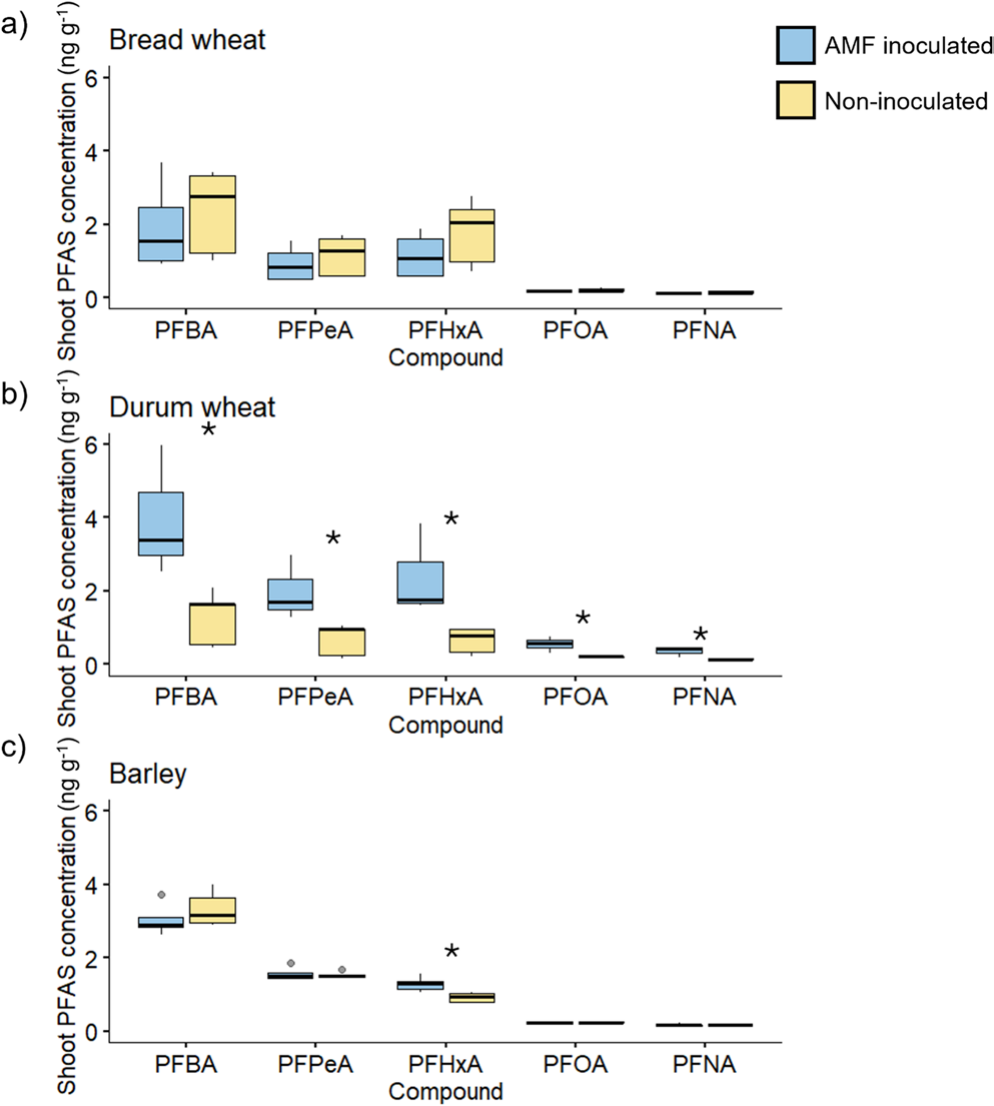
Concentration (ng g^− 1^) of four carboxylic PFAS in the shoots of bread wheat (**a**), durum wheat (**b**) and barley (**c**), grown in Soil 1 spiked with PFAS, and inoculated (blue) or not (yellow) with a commercial source of the AM fungus *Rhizophagus irregularis* (Experiment 2). Boxplots indicate the minimum, interquartile ranges, median (thick line), and maximum value, *n* = 5. Asterisks indicate a significant difference in treatment mean between the AMF-inoculated and non-inoculated plants for an individual sulfonic acid, following Student’s *t*-test

The plants from Expt. 2 were analysed for a subset of perfluourosulfonic (Fig. 5) and perfluorocarboxylic (Fig. 6) acid compounds. In general, the concentrations of the perfluorosulfonic acid compounds in durum wheat shoots in Expt. 2 were in the order of three times higher than in Expt. 1 (Fig. 3), but the shoot dry weights were comparable between experiments. Furthermore, in this experiment, the durum wheat plants had significantly higher concentrations of every measured compound of PFAS when colonised by AM fungi. For example, shoot PFBS concentration was mean 4.12 ng g^− 1^ in the inoculated plants, compared to 1.65 ng g^− 1^ in the non-inoculated plants, and PFBA concentration was mean 3.14 ng g^− 1^ compared with 1.27 ng g^− 1^.

The barley plants also accumulated three measured PFAS compounds (two sulfonic, one carboxylic) more when colonised by AM fungi: PFBS, PFPeS and PFHxA. The PFBS concentrations in barley shoots were 2.26 ng g^− 1^ compared with 1.8 ng g^− 1^.

In contrast to the other plant species, in bread wheat the concentration of PFAS compounds were generally lower than in durum wheat or barley, and there were no significant effects of AM colonisation on the concentration of any measured PFAS compounds.

## Discussion

In these experiments we empirically test the effect of AM fungi on the accumulation of various PFAS compounds in cereal plant tissues. Inoculation with AM fungi could have a: (1) neutral, (2) decreasing, or (3) increasing effect on shoot PFAS concentrations, and we tested this via two independent pot experiments that used non-sterilised soil (Expt. 1) or sterilised soil (Expt. 2); both experiments used the same soil, were spiked with PFAS in the same way, and included an inoculation treatment with a commercial source of *R. irregularis*.

### Cereal crops accumulate PFAS differently

In this study, the durum wheat shoots generally had higher concentrations of longer chain PFAS compounds than barley or bread wheat, especially of PFOA (CF_2_,7), PFOS and PFNA (CF_2_,8). On the other hand, barley shoots tended to have higher concentrations of short-chain (CF_2_, 4–6) PFAS compounds. Longer-chain PFAS compounds are less water soluble but have a greater tendency to bioaccumulate in biological tissues (Adu et al. 2023). Furthermore, longer-chain PFAS are present at much higher concentration than other PFAS in soils. Other studies also observed that crop species is a determinant of PFAS uptake. In a field study conducted near a fluorochemical manufacturing facility, Liu et al. (2017) reported that the total concentration of 12 PFAS in wheat grains was more than 11 times higher than in maize grains.

While PFAS concentrations varied between the two soil types, the sandy nature of both soils may make the PFAS more readily available and prone to leaching. Previous work has shown that PFAS can leach almost completely, up to 100%, from sandy soils, regardless of their initial PFAS concentrations (Kabiri et al. 2022). However, these results were obtained under specific conditions involving leaching tests where soil was in contact with water at a liquid-to-solid (L/S) ratio of 1:20, subjected to vigorous shaking for 24 h. Additionally, both our research and that of others have demonstrated that short-chain PFAS compounds leach more rapidly than long-chain PFAS in column leaching experiments. Due to their higher solubility and leachability, short-chain PFAS are also more bioavailable and more likely to be taken up by plants (Kabiri and McLaughlin 2021; Kabiri et al. 2022; Stahl et al. 2013). In general, the concentrations of sulfonic acids in the plants were similar to that of the carboxylic acids, even though sulfonic acids are less mobile in soil solution (Adu et al. 2023). This is likely because of the higher concentration of sulfonic acids in the soil (approximately 99% of PFAS measured in Soil 1 was PFOS and PFHxS).

The elevated accumulation of longer chain compounds in durum wheat in this study may be due to its inherently higher protein content compared to bread wheat and barley (García-Puebla et al. 2023; Zilić et al. 2011). In Poland, a survey was undertaken of 89 commercial food products including flours, bread, pasta and noodles purchased from supermarkets and analysed for ten PFAS compounds (Surma et al. 2022); the authors found that among the processed foods (i.e., not flours), the durum noodles (pasta) had a higher mean concentration of PFAS (5.6 ng g^− 1^) than all other products, including wheat noodles (3.17 ng g^− 1^). The tissue PFAS concentrations reached in our study were greater (by 2–3 orders of magnitude), but we measured PFAS in shoots rather than the food product where it would be diluted, and on much smaller plants than those the food products originated from.

More broadly, multiple studies have linked plant protein content with accumulation of PFAS compounds. In a plant study, it was found that root uptake and root-to-shoot translocation of PFOS and PFOA were positively correlated with plant protein content, so the legumes (soybean and mungbean) with higher protein contents, also had higher root concentration factors than the cereal crop ryegrass (Wen et al. 2016). More recently, Li et al. (2024 also found that soybean had the highest PFAS concentrations of the crops surveyed, including wheat and rice, reinforcing the correlation between protein content and PFAS accumulation in food crops.

The inclusion of a field contaminated soil in Expt. 1 served to highlight that PFAS accumulation in plants is not ubiquitous, and is highly dependent on the concentration and speciation of compounds in the soil, as well as soil chemical (Yang et al. 2024) and biological (Wu et al. 2023) properties that modify the solubility of the different compounds.

### AM fungal colonisation modulates shoot PFAS concentrations

The relationship between AM fungi and their host plant hinges on the transfer of soil-based resources from the fungus to the host plant (Smith and Read 2008). Generally, research into plant-AM fungal associations is focused on those resources that are necessary for plant growth and reproduction, such as a essential nutrients and water (Watts-Williams 2022). However, anthropogenic soil contamination streams such as heavy metals and microplastics have more recently come in to focus as evidence builds that AM fungi can ‘protect’ the host plant from accumulating contaminants in their aboveground tissues (Chen et al. 2023; Gonzalez-Guerrero et al. 2008; Riaz et al. 2021). While it has been shown that AM fungi can physically prevent the uptake of microplastics into the tissues of host plants (Chen et al. 2023), the limited published data on their relationship with plant PFAS uptake concurs with our findings, that AM fungal colonisation can increase plant PFAS uptake (Wang et al. 2025).

In this study, we found that inoculating durum wheat plants with *R. irregularis* led to an increase in the concentration of PFAS compounds in the shoots, in both sterile and non-sterile soils. This is in line with the results of Wang et al. (2025), who found that inoculation with *R. irregularis* doubled the uptake of some PFAS compounds in the model plant *I. pseudacorus*. This result was also observed to a lesser extent (i.e., in fewer of the measured PFAS compounds) in the barley plants. The contrasting lack of any effect of AM fungi on the concentration of PFAS compounds in bread wheat suggests that the interaction between AM fungi and PFAS concentration effect is crop-dependent. There was no effect of AM colonisation on shoot biomass in these experiments, making it unlikely that the observed effects on PFAS concentrations in durum and barley shoots were due to tissue dilution factors.

The effect of AM fungi on host plant PFAS accumulation may be direct (uptake by AM hyphae of PFAS from the soil and transport to plant across the peri-arbuscular membrane) or indirect (influence plant uptake via chemical modifications to the rhizosphere). On the other hand, the mechanisms of PFAS uptake by plants is incompletely understood. Some evidence suggests both passive and active transport pathways may be involved (Adu et al. 2023). Studies using metabolic and channel inhibitors indicate that PFOA and other PFAS can be taken up through energy-dependent processes, while PFOS uptake potentially involves aquaporins and anion channels. It has been proposed that wheat plants acquire PFAS from the soil by two mechanisms: ultra-short chain (CF_2_, 2 and 3) compounds that are highly water soluble could be taken up by aquaporins, while long-chain (CF_2_ ≥ 6) uptake is an energy-dependent active process (Zhang et al. 2019). Once there is more information available on the mechanism(s) for plant, and potentially fungal, PFAS uptake, it may help us predict which crop species may be more or less susceptible to tissue accumulation of PFAS.

### Soil PFAS contamination was not antagonistic to fungal or plant biomass

In these experiments, we did not observe any harmful effects of PFAS contamination on AM fungal colonisation of roots. In the case of barley, almost all roots were nearly 100% colonised by AM fungi including those exposed to PFAS in the spiked (with high concentration) and field-contaminated soils. This is in contrast to work that showed that AM fungal colonisation of spring onion roots was reduced in the presence of PFOS contamination in the soil (Yan et al. 2025). The difference was most likely due to the concentration of PFAS in the soil being around 10× higher in Yan et al. (2025) than in our study. Additionally, Yan et al. (2025) measured arbuscular and vesicular colonisation, while we quantified the broader measure of total colonisation, which may have overlooked a PFAS-dependent effect on abundance of arbuscules or vesicles specifically.

We also observed no negative impacts on plant biomass of spiking a soil with PFAS, even though the shoots accumulated very high concentrations of some of the PFAS compounds. The capacity for herbaceous plants to accumulate PFAS in large amounts or to high concentrations in aboveground tissues is what makes them excellent candidates for PFAS phytoextraction, and warrants further research (Huff et al. 2020; Kavusi et al. 2023).

## Conclusions

Here, shoot PFAS concentrations were influenced by the mycorrhizal status of the host plant, and the identity of the cereal crop. Of the three crops, durum wheat shoots consistently had higher concentrations of PFAS when colonised by AM fungi, supporting the hypothesis that AM fungi promote PFAS uptake of the host plant. In contrast, the effect of AM fungi was less, or not observed at all, in barley and bread wheat.

Future studies of this nature should focus on using soils with PFAS concentrations and speciation that is realistic in an agricultural context, such as those with historical biosolid application. There should also be a focus on growing crops to maturity in future experiments, so that the true potential transfer of PFAS in foods can be estimated.

## Supplementary information

Below is the link to the electronic supplementary material.

Supplementary Material 1 (DOCX 22.4 KB)

## Data Availability

All data are available upon reasonable request to the corresponding author.
